# Isolation of infectious SARS-CoV-2 from urine of a COVID-19 patient

**DOI:** 10.1080/22221751.2020.1760144

**Published:** 2020-05-18

**Authors:** Jing Sun, Airu Zhu, Heying Li, Kui Zheng, Zhen Zhuang, Zhao Chen, Yongxia Shi, Zhaoyong Zhang, Si-bei Chen, Xuesong Liu, Jun Dai, Xiaobo Li, Shuxiang Huang, Xiaofang Huang, Ling Luo, Liyan Wen, Jianfen Zhuo, Yuming Li, Yanqun Wang, Lu Zhang, Yanjun Zhang, Fang Li, Liqiang Feng, Xinwen Chen, Nanshan Zhong, Zifeng Yang, Jicheng Huang, Jincun Zhao, Yi-min Li

**Affiliations:** a State Key Laboratory of Respiratory Disease, National Clinical Research Center for Respiratory Disease, Guangzhou Institute of Respiratory Health, the First Affiliated Hospital of Guangzhou Medical University, Guangzhou, People’s Republic of China; b State Key Laboratory of Respiratory Disease, Guangzhou Institutes of Biomedicine and Health, Chinese Academy of Sciences, Guangzhou, People’s Republic of China; c Guangzhou Customs District Technology Center, Guangzhou, People’s Republic of China; d Institute of Infectious disease, Guangzhou Eighth People’s Hospital of Guangzhou Medical University, Guangzhou, People’s Republic of China

**Keywords:** COVID-19, urine, transmission, SARS-CoV-2, virus isolation

## Abstract

SARS-CoV-2 caused a major outbreak of severe pneumonia (COVID-19) in humans. Viral RNA was detected in multiple organs in COVID-19 patients. However, infectious SARS-CoV-2 was only isolated from respiratory specimens. Here, infectious SARS-CoV-2 was successfully isolated from urine of a COVID-19 patient. The virus isolated could infect new susceptible cells and was recognized by its’ own patient sera. Appropriate precautions should be taken to avoid transmission from urine.

## Letter

A novel coronavirus SARS-CoV-2 emerged to cause a major outbreak of severe pneumonia in humans in China and has spread to over 100 other countries [1]. As of April 6th 2020, 1,210,956 cases with 67,594 deaths (5.6% case fatality rate) had been reported to the World Health Organization (WHO). The major transmission routes are believed to be exposure to virus-containing droplets or contaminated fomites [2]. Although viral RNA can be detected in multiple organs in COVID-19 patients, infectious SARS-CoV-2 has only been isolated from respiratory specimens [3,4]. Since the viral receptor, angiotensin-convertase-2 (ACE2) protein, is expressed in the kidney, the testis and the bladder [5,6] and viral RNA has been detected in the sink and toilet of COVID-19 patients, the virus may be secreted through the urinary system [7]. However, it is currently unclear if infectious virus is secreted in urine.

**Figure 1.**
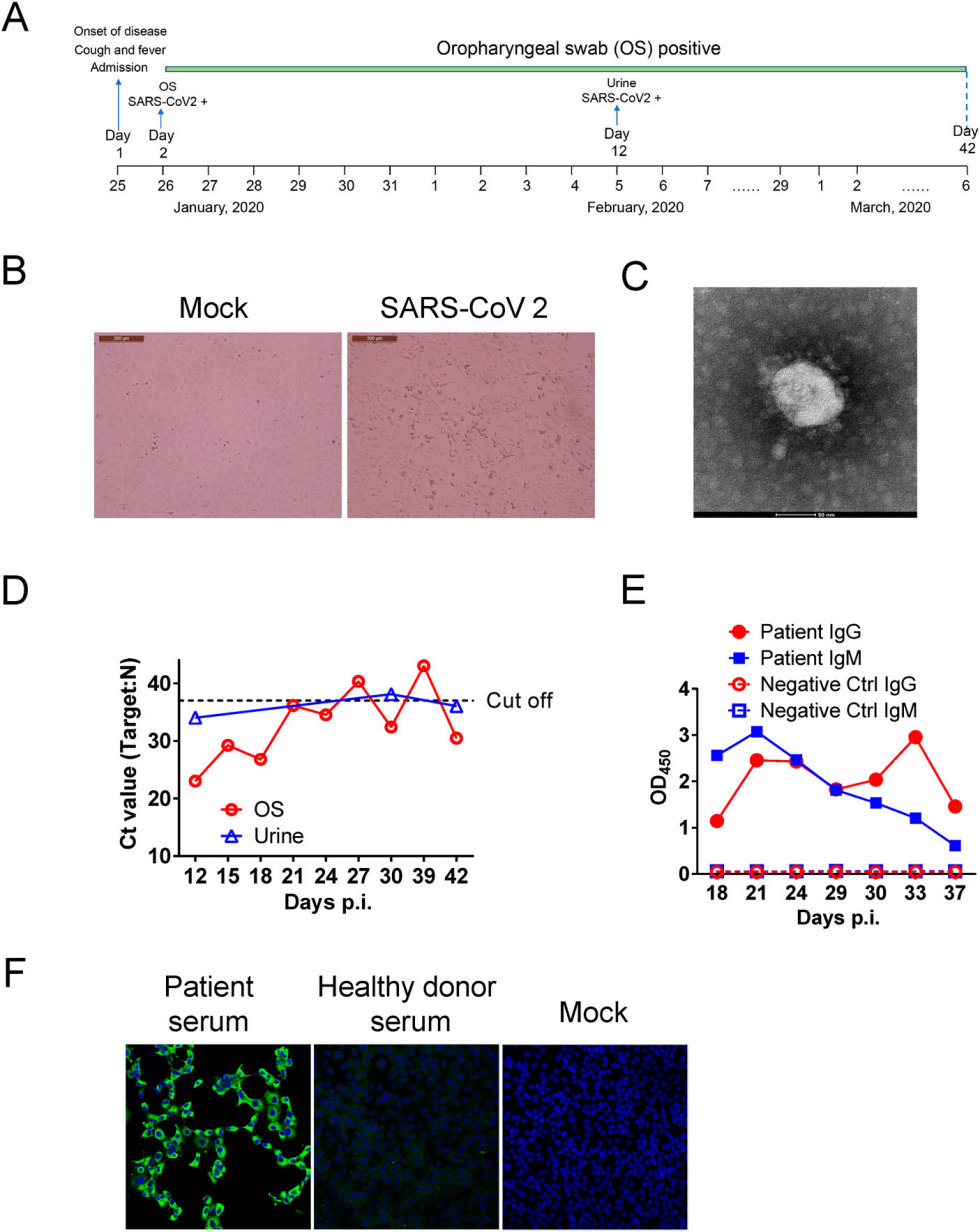
Clinical information and isolation of SARS-CoV-2 from a patient’s urine. (A) Clinical events. (B) Cytopathic effect (CPE) were observed in Vero E6 cells that were infected with SARS-CoV-2 isolate after 72 h but not in mock-infected cells. (C) Visualization of viral particles using Transmission Electron Microscopy (TEM). (D) Viral loads in respiratory and urine specimens. OS: oropharyngeal swab. (E) SARS-CoV-2-specific IgG and IgM antibody responses in patient. (F) IFA detection of SARS-CoV-2 infected Vero E6 using patient’s serum. No fluorescence was detected using healthy control serum or in mock-infected cells.

On 25 January 2020, a 72-year-old man who had a history of recent travel to Wuhan was admitted to the Eighth People's Hospital of Guangzhou Medical University with cough and fever. He was then transferred to the First Affiliated Hospital of Guangzhou Medical University (Figure 1A). Oropharyngeal swabs were positive for SARS-CoV-2 RNA by RT–PCR on January 26th. Computed tomography (CT) scan of the chest revealed multiple ground-glass opacities in both lungs as shown in Figure S1. On February 3rd, the patient’s condition deteriorated and he was intubated and put on a ventilator. The urine sample tested positive for SARS-CoV-2 RNA on day 12 post infection (p.i.) (February 5th) for the first time and had periodically showed positive results in RT–PCR test until March 6th.

RT–PCR positive urine specimens (Ct 34) from day 12 p.i. was serially diluted in infection media and inoculated onto Vero E6 cells. Cytopathic effects (CPE) were clearly observed after 3 days (Figure 1B). Full-length viral genome sequence was acquired by next-generation sequencing (Accession number MT446312) with 5 nucleotide mutations as compared to the original Wuhan viral stain (Accession number NC045512.2) (Table S1). Cell culture supernatant was negatively stained and visualized by transmission electron microscopy (TEM). Spherical-shaped particles with distinct surface projections, resembling spikes, that were consistent with SARS-CoV-2 virions as published previously (Figure 1C) were observed [1]. Viral load in respiratory specimens and urine were quantitated by RT–PCR. Viral loads decreased over time in oropharyngeal swabs. Viral load in urine was low but detectable at day 12 and day 42 p.i., but not at day 30 (Figure 1D). The patient had a high level of SARS-CoV-2-specific IgM at early time points and declined over time while the IgG antibodies remained relatively stable (Figure 1E). In order to prove that the isolated virus was infectious to susceptible cells, fresh Vero E6 cells was inoculated with the virus and stained in an immunofluorescent assay (IFA) using serum obtained from the patient and from a healthy donor. Results showed positive fluorescence with patient’s serum but not with healthy control serum (Figure 1F).

Although it is hard to determine whether the kidney, the testis or the bladder were infected and produced infectious virus from current study, isolation of infectious SARS-CoV-2 in urine raises the possibility of fecal/urine-respiratory transmission. In the 2003 Severe Acute Respiratory Syndrome (SARS) epidemic, 329 patients were infected resulting in 42 deaths in a community called Amoy Gardens in Hong Kong [8]. Investigation of the building structure showed that faulty sewage pipelines led to aerosolization of feces and urine contaminated with SARS-CoV, which infected almost 300 people in the apartment complex. These findings raise the importance of using appropriate precautions to avoid transmission from urine.

## Contributors

J.Z., Y.L., J. H. and Z.Y. conceived the study, J.S., A.Z., H.L., Z.Z., Z.C., Z.Z., S.C., L.X. collected clinical specimen and executed the experiments, and all of the authors participated in data analysis. J.Z., J.S. wrote the manuscript.

## Supplementary Material

Supplemental Material
