# Supplementary material for: Isolation of infectious SARS-CoV-2 from urine of a COVID-19 patient
Source: Emerg Microbes Infect. 2020 May 18;9(1):991–3. doi: 10.1080/22221751.2020.1760144 (PMC7301718; doi:10.1080/22221751.2020.1760144)
Supplement: Supplemental Material [file TEMI_A_1760144_SM2136.docx]

**SUPPLEMENTAL Appendix**

**Isolation of Infectious SARS-CoV-2 from urine of a COVID-19 Patient**

**Running title:** Infectious SARS-CoV-2 in patient’s urine

Jing Sun^1^*, Airu Zhu^1^*, Heying Li^2^*, Kui Zheng^3^*, Zhen Zhuang^1^*, Zhao Chen^1^*, Yongxia Shi^3^*, Zhaoyong Zhang^1^*, Si-bei Chen^1^*, Xuesong Liu^1^*, Jun Dai^3^, Xiaobo Li^3^, Shuxiang Huang^3^, Xiaofang Huang^1^, Ling Luo^1^, Liyan Wen^1^, Jianfen Zhuo^1^, Yuming Li^1^, Yanqun Wang^1^, Lu Zhang^1^, Yanjun Zhang^1^, Fang Li^1^, Liqiang Feng^2^, Xinwen Chen^2^, Nanshan Zhong^1^, Zifeng Yang^1#^, Jicheng Huang^3#^, Jincun Zhao^1,4#^, Yi-min Li^1#^

^1^State Key Laboratory of Respiratory Disease, National Clinical Research Center for Respiratory Disease, Guangzhou Institute of Respiratory Health, the First Affiliated Hospital of Guangzhou Medical University, Guangzhou, China 510182

^2^State Key Laboratory of Respiratory Disease, Guangzhou Institutes of Biomedicine and Health, Chinese Academy of Sciences, Guangzhou, China 510530

^3^Guangzhou Customs District Technology Center, Guangzhou, China 510700

^4^Institute of Infectious disease, Guangzhou Eighth People's Hospital of Guangzhou Medical University, Guangzhou, China 510182

*Contributed equally to this manuscript

^#^Corresponding authors: Dr. Jincun Zhao, [zhaojincun@gird.cn](mailto:zhaojincun@gird.cn)

Dr. Yimin Li, dryiminli@vip.163.com

Dr. Jicheng Huang, [jichenghuang@126.com](mailto:jichenghuang@126.com)

Dr. Zifeng Yang, jeffyah@163.com


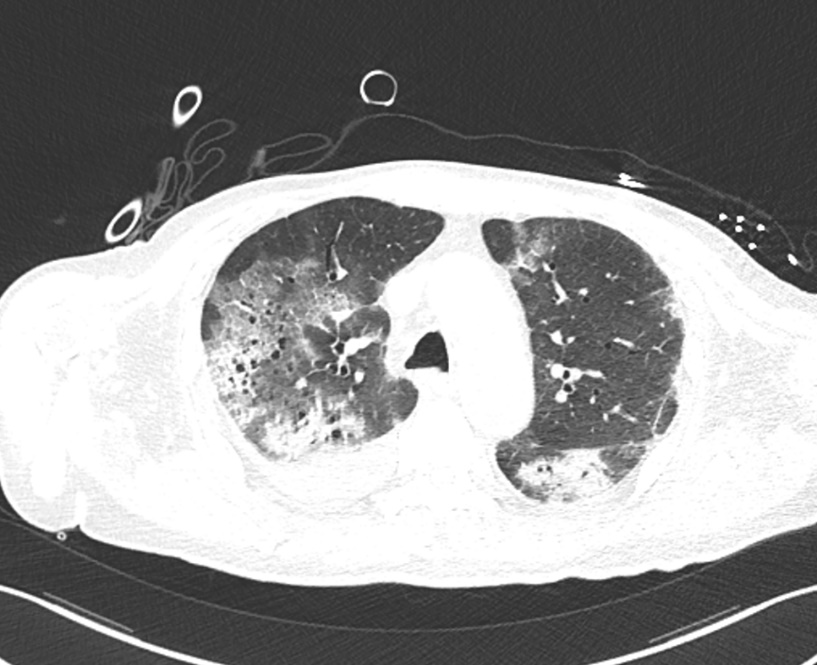


**Figure S1. Chest CT scan of the SARS-CoV-2 infected patient**

**Table S1. Nucleotide and amino acid changes between original virus strain (Wuhan-Hu-1 NC_045512.2) and the virus isolated from the patient’s urine.**

| Genome nucleotide position | Protein | Nucleotides changes | | Amino acides changes |
| --- | --- | --- | --- | --- |
|  |  | NC_045512.2 | Urine SARS-CoV-2 |  |
| 6819 | ORF1ab | G | T | Ser>Ile |
| 6996 | ORF1ab | T | C | Ile>Thr |
| 14235 | ORF1ab | C | T | Thr>Ile |
| 17373 | ORF1ab | C | T | Synonymous |
| 29527 | N | G | A | Synonymous |
